# Surgeon Learning Curve and Clinical Outcomes of Minimally Invasive Anterior Lumbar Interbody Fusion With Posterior Percutaneous Instrumentation

**DOI:** 10.5435/JAAOSGlobal-D-22-00207

**Published:** 2022-12-05

**Authors:** M. Zain Mirza, Sydney L. Olson, Annalise M. Panthofer, Jon S. Matsumura, Seth K. Williams

**Affiliations:** From the Department of Surgery (Ms. Olson, Ms. Panthofer, and Dr. Matsumura) and Department of Orthopedics and Rehabilitation (Dr. Mirza and Dr. Williams), University of Wisconsin School of Medicine and Public Health, Madison, WI.

## Abstract

This study reports the learning phase of the minimally invasive anterior lumbar interbody fusion (mini-ALIF) approach with a vascular and orthopaedic spine surgeon team.

**Methods:**

Adult patients who underwent primary mini-ALIF at the lowest two segments of the lumbar spine (i.e., L4/5, L5/S1) between January 2010 and December 2018 were analyzed.

**Results:**

One hundred twenty-seven patients were included. There was no notable change in total surgical time over the study period. Estimated blood loss markedly decreased until stabilizing at case 30 and slowly declined thereafter. The mean estimated blood loss was 184 mL for L5/S1, 232 mL for L4/L5, and 458 mL for two-level mini-ALIF. There were 20 vascular issues requiring primary repair or packing. Vascular issues declined over time, with a rate of 32% in the first 25 cases and 0% in the last 25. The postoperative complication rate was highest in the first 25 cases (7 of 21 total complications). The odds ratio of vascular injury with body mass index (BMI) > 35 was 4.09 (1.4 to 11.7 confidence interval, *P ≤* 0.008). Total surgical time and postoperative complications increased with increasing BMI.

**Conclusion:**

Performance of the mini-ALIF approach is associated with a learning curve of 25 to 30 cases before complications begin to decline. BMI > 35 is associated with increased surgical time and complications.

The anterior lumbar approach was first introduced in the 1930s by Capener and Ito et al as a treatment of spondylolisthesis and Pott disease.^1,2^ Since then, multiple variations have been introduced, including open and laparoscopic transperitoneal, open and endoscopic retroperitoneal, and mini-open retroperitoneal.^3^ Harmon first described the retroperitoneal approach in 1963, which was later modified by Mayer into a minimally invasive approach.^3^ Lumbar interbody fusion was initially described by Briggs in 1944.^4^ A notable increase has been observed in minimally invasive anterior and anterolateral approaches to the lumbar spine over the past 2 decades including mini-open anterior lumbar interbody fusion (mini-ALIF), transpsoas lateral lumbar interbody fusion, and the anterior-to-psoas oblique lateral approach.^5^ Depending on the patient's underlying pathology, the goals of these fusion procedures may include correction of scoliosis and/or sagittal malalignment, spondylolisthesis reduction, and/or indirect neural element decompression while minimizing posterior paraspinal muscle dissection, blood loss, and pseudarthrosis rates.

Historically, ALIF was conducted through a relatively large extensile procedure, but Brau^6^ popularized the mini-open retroperitoneal lumbar spine approach technique in the early 2000s, which uses substantially smaller incisions that are just big enough to provide a working corridor to conduct the fusion. The mini-ALIF approach, with an incision in the anterior left lower quadrant spanning a length of <5 cm, allows for a direct midline exposure of the lumbar spine, permitting a wide diskectomy and placement of an implant with a relatively large end plate footprint when compared with posterior interbody fusion techniques. Implant placement with disk height restoration and/or spondylolisthesis reduction produces indirect decompression of the neural elements while minimizing paraspinal muscle damage.^7,8^ Radiographic studies have shown a 67% increase in the foraminal cross-sectional area after an ALIF. For lateral lumbar interbody fusion and anterior-to-psoas lateral fusion, this increase is reported to be 24.7% to 30.0%.^5^ Moreover, ALIF cages have a relatively low subsidence rate because of the size of the end plate footprint, low overall blood loss when compared with posterior techniques, and better radiographic outcomes.^5,9,10^

Although it does have advantages, ALIF is associated with potentially life-threatening vascular complications. These complications predominantly occur while obtaining anterior access to the spine because of the proximity of the major blood vessels to the target disk space (iliac artery and vein, aorta, and inferior vena cava). Rates of vascular injury reported in the literature range from 1.9% to as high as 15%.^11–13^ Given the potentially life-threatening risk associated with the ALIF approach, it is imperative that surgeons are well-trained and familiar with the retroperitoneal approach. Although similar studies have been conducted for other minimally invasive spine procedures, the surgeon learning curve for anterior lumbar interbody fusions with posterior percutaneous instrumentation is not well-studied.^14^ The purpose of this study was to report the surgeon learning curve and complications associated with the initial learning phase of the mini-ALIF approach.

## Methods

### Overview

Data were retrospectively collected for patients who underwent anterior lumbar interbody fusion from first use of the minimally invasive technique in January 01, 2010, through December 31, 2018, in the University of Wisconsin (UW) system (UW Hospitals and Clinics and UW Health at the American Center). Patients were identified by querying Current Procedural Terminology (CPT) code 22558 (arthrodesis, anterior interbody technique, including minimal diskectomy to prepare the interspace) for the vascular surgeon, which was the only anterior lumbar spine approach conducted by the vascular surgeon. All patients underwent the anterior approach by the same vascular approach surgeon, followed by disk preparation and implant placement by one of three fellowship-trained spine surgeons. This study was approved by the Health Sciences Institutional Review Board at the UW (IRB ID: 2017 to 1274), and informed consent was waived.

### Inclusion and Exclusion Criteria

**Figure 1:**
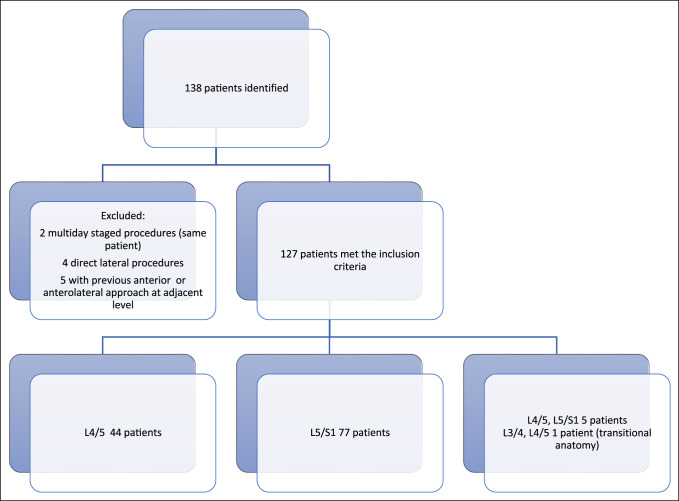
Graph showing subject inclusion.

Patients 18 years or older undergoing a primary anterior approach for anterior fusion of the lumbar spine (i.e., L4/5, L5/S1) were included in this study. Patients with a previous anterior or anterolateral approach at adjacent levels were excluded. Patients who underwent anterolateral or lateral interbody procedures were also excluded (Figure 1). One patient who underwent a multiday staged procedure was also excluded. Of 138 patients, 127 patients met the inclusion criteria. Forty-four patients underwent a L4/5 fusion; 77 patients underwent a L5/S1 fusion; five patients underwent a two-level fusion (L4/5, L5/S1); and one patient with transitional anatomy underwent a lowest mobile segment two-level fusion (L3/4, L4/L5). Preoperative diagnoses included degenerative spondylolisthesis, degenerative disk disease, isthmic spondylolisthesis, spondylolysis, lumbar stenosis, recurrent disk herniation, and pseudarthrosis.

### Data Collection

The following data were collected through an extensive medical record search for the 127 patients who met the inclusion criteria: age at the time of surgery, sex, body mass index (BMI), diagnosis, common comorbidities (diabetes, peripheral vascular disease, nicotine use), intraoperative estimated blood loss (EBL) for the entire procedure including any posterior component, intraoperative vascular issues requiring even minor repair or packing, duration of procedure (anterior, posterior, total), infection, hematoma, deep vein thrombosis (DVT), hospital length of stay (LOS), and 30-day readmission rates. EBL was calculated by consensus between surgeons and anesthesiologists at the end of the case; intraoperative blood salvage was not routinely used. Total surgical time was defined as the time of incision for the anterior portion until the time of closure of the posterior percutaneous incisions; these times were collected as charted in the patient records.

### Surgical Technique

When both anterior and posterior procedures were done during the same surgical event, the anterior component was completed first. The anterior retroperitoneal approach was similar to the mini-open approach described by Brau.^6,11^ A preoperative review of the spinal and vascular anatomy is done to identify anatomic variants. For example, higher and lower venous and arterial bifurcations are common, whereas aberrant origin of the hypogastric veins and left-sided inferior vena cava are rarer. More difficult exposure can be anticipated by being mindful of large osteophytes and high-grade spondylolisthesis which stretch the overlying vessels. Intrinsic vascular pathology such as atherosclerotic calcifications, thrombotic venous disease, and previously implanted stents are important to identify. Rarely, a pelvic kidney or other pertinent developmental aberrant anatomy is diagnosed. Prior surgical notes and radiation therapy markers are reviewed. A gentle bowel prep is prescribed, typically consisting of oral Dulcolax the morning of the day before surgery.

Patients are positioned supine with the hips and knees flexed approximately 30° and supported by pillows under the knees, to take tension off the psoas muscles, and the arms folded above the chest. Fluoroscopy is used to identify the level of interest and assist with incision location, and then, an oblique skin incision is made (<5 cm) over the left side of the abdomen with the approach surgeon standing on the patient's left side. Tunneling throughout all layers is kept limited, with the expressed goal of creating a cylindrical tunnel of approximately 5 cm in diameter from the surface down to the anterior surface of the disk space in direct alignment with the midline and the native lordosis. Handheld retractors are used to allow immediate shifting of this tunnel to optimize visualization of the parts of the disk space being actively worked on. This also permits relaxation of vascular retraction from time to time, permitting intermittent unimpeded flow in the left lower extremity.

The dermis and lateral rectus sheath are infiltrated with 0.25% bupivacaine. After subcutaneous incision down to the anterior rectus sheath, subcutaneous flaps are mobilized beyond the edges of the incision and a vertical incision made in the rectus sheath. Often, a lateral extension at the cranial end is added to the rectus sheath incision. The anterior rectus sheath is then elevated from the muscle belly to mobilize the rectus muscle until the lateral edge of the muscle is identified. This is retracted medially along with the inferior epigastric vessels on its undersurface. The posterior rectus sheath is variable in thickness, and when substantial, it is identified and bluntly dissected from the peritoneum with rotating finger motion to avoid entry into the underlying peritoneum. Next, a plane within the retroperitoneal fat is identified, sweeping most of the fat medially until the left psoas muscle is identified. The peritoneal contents are then further retracted medially to expose the iliac vessels posteriorly. The left ureter is identified, mobilized anteriorly and medially, and protected. Handheld retractors in combination with bipolar cautery are used to both retract and dissect in these tissue planes.

Left external and common iliac arteries are exposed and the left common iliac vein identified deep to it. For L4/5 procedures, the iliolumbar vein is usually identified traversing posteriorly into the left paraspinous area, is ligated in a secure manner with silk ties and a knot pusher, and is divided to help with mobilization of the iliac vein. Often, additional segmental branches are ligated and divided for further mobilization. Occasionally, ample slack is available so that mobilization does not require division of these branches. After this, the left common iliac vein and artery are carefully dissected off the L4/5 annulus and gently retracted with handheld retractors throughout the whole case.

For L5/S1 access, dissection is done anteromedial to the left iliac vein. Middle sacral vessels are ligated and divided. At this time, iliac veins are mobilized and protected with handheld retractors as needed for the remainder of the procedure. For notable anterolisthesis or osteophytes that prevent full initial mobilization of vessels before disk preparation, a stepwise meticulous vessel dissection and disk preparation and reduction are done alternating back and forth between the two steps until adequate exposure is obtained for safe interbody cage implantation.

### Statistical Analysis

Logarithmic regression was used to assess the learning curve by identifying trends in surgical time, EBL, vascular injury, and other complications.^15^ For complications associated with higher BMI, odds ratio was calculated and *P* < 0.05 was considered statistically significant. MedCalc Stat software was used for statistical analysis.

For multilevel cases, the mean surgical time was corrected with a coefficient K of 1.20, which was derived by dividing mean surgical time for L5/S1 by mean surgical time for L4/5 and L5/S1 (Table 1); this correction was used to allow comparison of multilevel cases with a single level in monitoring surgeon growth curves.

**Table 1:** Mean Total Surgical Time, Intraoperative Vessel Issues, Postoperative Complications, Mean BMI, and Mean EBL

	n	Mean Surgical Time (min)	Intraoperative Vessel issues	Postoperative Complications n, (%)	Mean BMI	Mean EBL (mL)	Coefficient K
L5/S1 ALIF	77	228	12	18, (23%)	29.9	184	1
L4/L5 ALIF	44	228	7	9, (20%)	29.7	232	1
Two-level ALIF	6	274	1	1, (17%)	26.3	458^a^	1.20

BMI = body mass index, EBL = estimated blood loss

a One patient had transitional anatomy and lost 1,800 mL because of vessel injury.

## Results

### Patient Demographics

Of the 127 patients identified, the mean age was 52 years (range 24 to 84), with 55 males and 72 females. Most of the patients were American Society of Anesthesiologists (ASA) class 2 or 3 (96, 51, respectively). The mean BMI was 29.6 with a range from 17.2 to 49.3. Patient diagnoses are summarized in Tables 2 and 3. Case volume increased annually as the program grew and comfort and safety of the mini-ALIF approach increased; 3 cases were completed from 2010 to 2011, five from 2012 to 2013, 15 from 2014 to 2015, 56 from 2016 to 2017, and 48 in 2018.

**Table 2:** Diagnosis

Diagnosis	
Lumbar stenosis (Central/foraminal)	20
Spondylolisthesis (isthmic and degenerative)	66
Retrolisthesis	3
DDD with or without radiculopathy	27
Recurrent HNP	3
Pseudarthrosis	6
Deformity	2

DDD = degenerative disk disease, HNP = herniated nucleus pulposus

**Table 3:** Baseline Characteristics

	Baseline Characteristic
Age	24–84 (52 mean, 52 median)
Sex	72 females, 55 males
BMI	17.2–49.3 (mean 29.6, SD 5.7)
ASA class	1:10 2:96 3:18 Unknown: 3
Smoker	Current: 9 Former: 61 Never: 57
Diabetes mellitus	8 patients

BMI = body mass index

### Surgical Time

**Figure 2:**
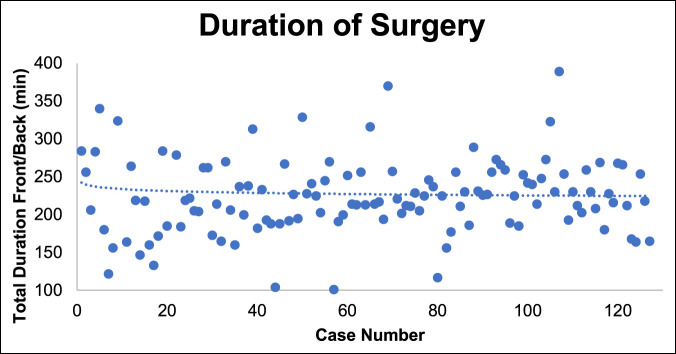
Graph showing the total surgical time of all mini-ALIF cases over the study period. mini-ALIF = minimally invasive anterior lumbar interbody fusion

**Figure 3:**
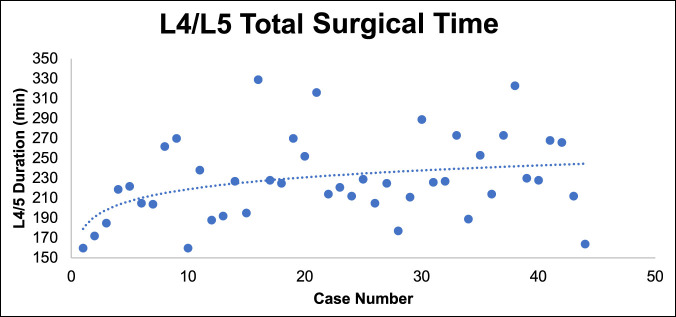
Graph showing the total surgical time of L4/L5 mini-ALIF cases over the study period. mini-ALIF = minimally invasive anterior lumbar interbody fusion

No significant decrease was observed in total surgical time over the 127 cases (Figure 2). When separating L4/5 and L5/S1 cases, we also did not find any notable decrease in surgical time for either level, although this may be confounded by the relative increase in complexity of cases over time. In fact, L4/5 total surgical time slightly increased and plateaued around case 25 (Figure 3).

Thirty-eight of 44 L4/L5 and 54 of 77 L5/S1 cases had surgical duration data separating anterior and posterior portions of the case. Within these patients, the time spent on the anterior portion of the case for both L4/5 and L5/S1 cases increased and plateaued around case 25.

### Estimated Blood Loss

**Figure 4:**
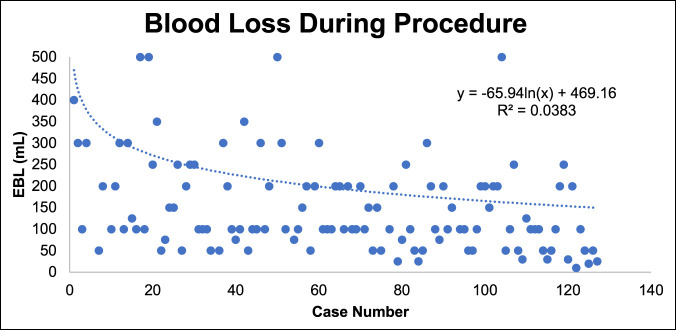
Graph showing EBL over time. Values more than 500 mL used in log regression fit calculation but not shown in a magnified chart for clarity. EBL = estimated blood loss

Total blood loss during the surgery significantly decreased until around case 30 before stabilizing with a slow decline over time (Figure 4). The mean EBL was 184 mL for L5/S1, 232 mL for L4/L5, and 458 for two-level ALIFs. One patient with transitional anatomy and a low bifurcation who underwent ALIF at the lowest two levels (L3/4, L4/5) had an intraoperative vascular injury with 1,800-mL EBL.

### Vascular Issues

**Figure 5:**
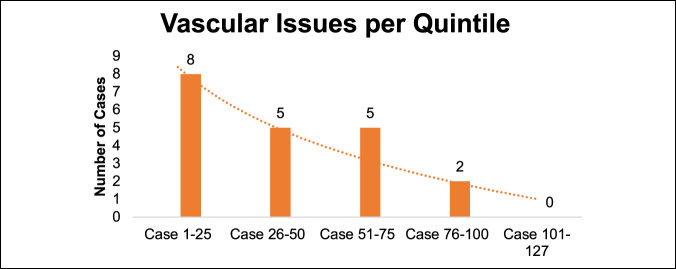
Graph showing vascular issues per mini-ALIF case quintile. mini-ALIF = minimally invasive anterior lumbar interbody fusion

Thirty-one potential vascular issues were recognized on initial chart review. After detailed analyses, 11 were planned ligation for safe mobilization and 20 were unplanned requiring either a primary repair or packing. The 20 unplanned issues were further divided into 12 (9.4%) involving major vessels (iliac vessels or inferior vena cava) and 8 (6.3%) involving small branches. Three of 20 issues were arterial (two iliac arteries and one arterial branch). We observed a decline in vascular issues that required a repair or packing over the course of this study with the highest rate occurring in the first quintile (25 of 127) (32%) versus zero percent in the last (Figure 5).

### Postoperative Complications

Postoperative complications are summarized in Table 4. No postoperative complications were observed in eight patients with diabetes or one patient with peripheral vascular disease.

**Table 4:** Summary of Complications

Complication	No. of patients
30-day readmission/ED visits	14 (11%)
DVT	3 (2.4%)
Delayed return of bowel function	3 (2.4%)
Lower extremity swelling, pain, and cramps	6 (4.7%)
Superficial wound complication (infection or hematoma)	7 (1 retroperitoneal hematoma) (5.5%)
Deep infection	1 (0.8%)
UTI	1 (0.8%)

DVT = deep vein thrombosis, UTI = urinary tract infection

**Figure 6:**
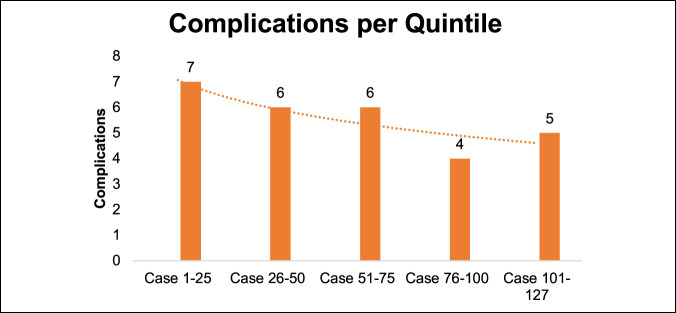
Graph showing complications per mini-ALIF case quintile. mini-ALIF = minimally invasive anterior lumbar interbody fusion

The number of complications over time is presented in Figure 6. Five of the nine current and 11 of 61 former (18%) smokers experienced a postoperative complication. Twelve of 57 patients (21%) who never smoked experienced complications.

### Body Mass Index

**Figure 7:**
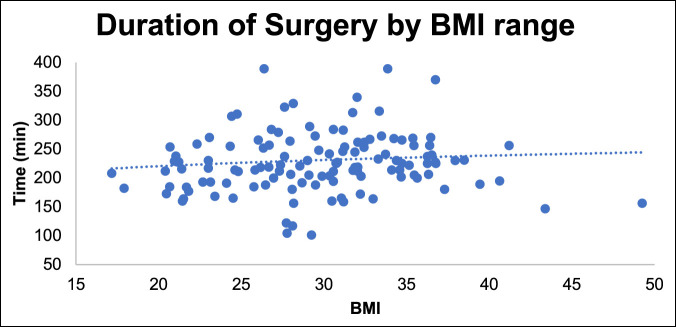
Graph showing duration of surgery by BMI. BMI = body mass index.

**Figure 8:**
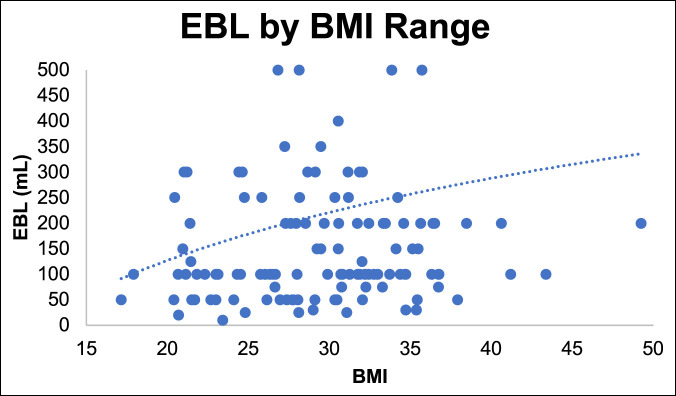
Graph showing the association of EBL with BMI. BMI = body mass index, EBL = estimated blood loss

**Figure 9:**
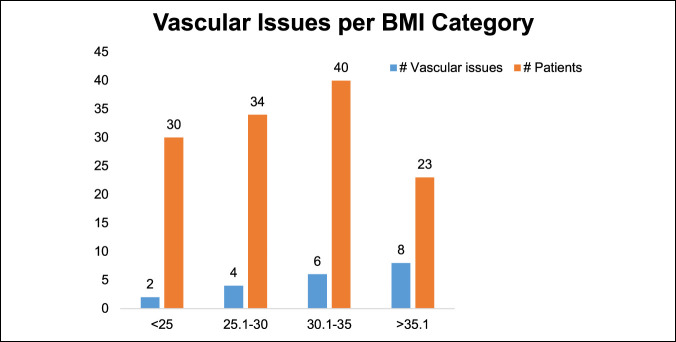
Graph showing the association of BMI with vascular issues. BMIs >35 report the highest rate of vascular complications. BMI = body mass index

We observed a longer duration of surgery, increased EBL, and increased rate of vascular issues with increasing BMI. There was a trend toward increased surgical time with increasing BMI (Figure 7) and increased blood loss (Figure 8). There were 12 vascular issues with BMI less than 35 (12%, n = 104 patients) and 8 with BMI greater than 35 (35%, n = 23 patients). The odds ratio of vascular injury with BMI more than 35 was significant at 4.09 (1.4 to 11.7 confidence interval [CI], *P* ≤ 0.008) (Figure 9).

**Figure 10:**
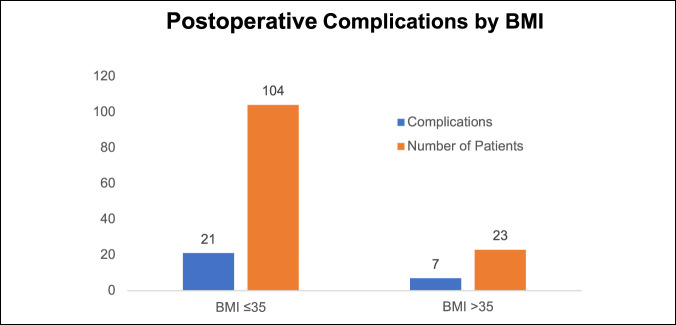
Graph showing the association of BMI with all complications. BMIs >35 report the highest rate of all complications. BMI = body mass index

The postoperative complication rate was 20.2% (21 of 104) with a BMI of 35 or less and 30.4% (7 of 23) with BMI greater than 35. This was not statistically significant, with an odds ratio of 1.73 (0.63 to 4.7 CI with a *P* value of 0.287) (Figure 10).

### Hospital Stay

**Figure 11:**
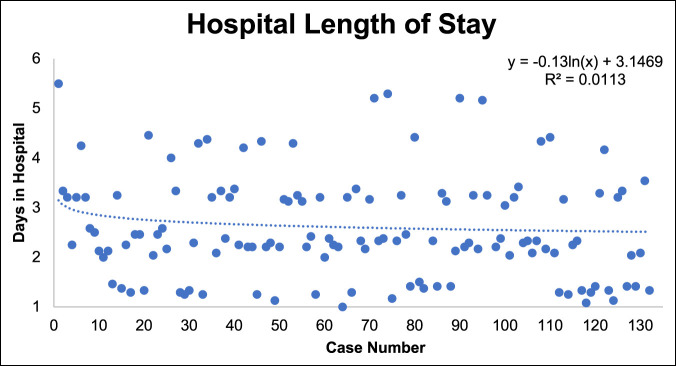
Graph showing hospital LOS over the study period. LOS = length of stay

The mean hospital stay was three days. No difference was observed in mean LOS between the L4/5 and L5/S1 groups. After initial decline, there was no significant reduction in hospital stay over the course of this study. (Figure 11).

## Discussion

From its first introduction by Capener and Ito in the 1930s to later modifications by Harmon, Mayer, and Brau, the anterior lumbar approach has evolved into a minimally invasive muscle-sparing technique for lumbar spine fusion.^1–3,6^ The use of the anterior retroperitoneal approach for lumbar spine fusion has increased in recent years because surgeons have recognized the benefits of a minimally invasive (muscle-sparing) approach to the spine that allows placement of an interbody cage with a large footprint.^7,8^ With the advent of percutaneous pedicle screw placement techniques over the past decade, the combination of a mini-ALIF approach with indirect decompression of the neural elements and posterior percutaneous instrumentation has allowed for effective treatment of radicular symptoms and spine stabilization with minimal morbidity, better fusion rates, improved radiographic outcomes, and a lower incidence of adjacent segment disease.^10,16,17^ The goal of this study was to analyze the surgeon learning curve as it relates to surgical duration, vascular issues, blood loss, and complication rates.

Procedure time, as a primary parameter, has been used by other authors evaluating learning curves in spine surgery.^14,18–20^ A 23% to 58% decline in surgical time has been reported with asymptote reaching around the 30th case.^14,18–20^ Our study showed an increase in surgical duration for the anterior portion of the case over time, which is likely multifactorial. Early in the series, the vascular approach surgeon personally conducted the approach, assisted by vascular surgery residents or fellows. This transitioned to the approach surgeon working exclusively with spine fellows during the approach, with the goal of training the spine fellows to safely conduct the approach, and this is likely the primary explanation for the increase in surgical time. Increased time was also possibly related to increased case complexity (i.e., larger BMI, complex vascular anatomy) as more confidence developed with the mini-ALIF approach over the study period, which meant that more complicated approaches were now being treated with the mini-ALIF approach rather than an all-posterior approach.

Sclafani et al reported an 11% overall postoperative complication rate in their systematic review of literature discussing different MIS learning curves ranging from microdiskectomies to laparoscopic ALIF. They noted that overall surgical time and complications reduced around 20 to 30 consecutive cases for most techniques.^14,21^ Our data showed that the anterior approach for lumbar interbody fusion is associated with a learning curve. Like other studies that looked at different minimally invasive spine techniques, approximately 25 cases are needed before the intraoperative and postoperative complication rates begin to drop.^14,21^ Improvements continued through the first 100 cases. Beyond 100 cases, BMI > 35 best predicts complication risk. One key point that we think helps limit complications and retractor readjustments and allows adequate intraoperative visualization is to precisely plan the incision and, therefore, the surgical trajectory using fluoroscopy. The mini-ALIF approach uses an incision that is just long enough to establish the smallest possible working corridor to safely conduct the surgery, so surgical instruments are most effective when the trajectory from the incision to the disk is in line with the disk space. Multiple authors have asserted the importance of correct initial incision planning and trajectory while conducting MIS techniques in general.^14,22–24^

Teng et al^10^ reported equal fusion and complication rates in ALIF versus TLIF versus posterior lumbar interbody fusion, but superior radiographic outcomes in ALIF patients, along with the least amount of blood loss in their meta-analysis (200 to 300 mL average for the ALIF). They cautioned that the EBL could easily double in the case of a vascular injury. This was consistent with our results of 184 mL (L5/S1) and 232 mL (L4/L5) mean EBL. Although ALIF is associated with the least amount of blood loss when compared with other techniques, it has the potential to rapidly turn into a life-threatening event with rapid blood loss as evidenced by one patient with approximately 1800 mL EBL in our study because of transitional anatomy.

Vascular injury can be a potentially devastating complication during an ALIF. The rate of major vessel injury (12 of 127) in our study was 9.4% and consistent with published studies that quoted 1% to 24%.^6,11–13,25^ Most of the reported vascular issues are venous with 0.45% to 1.5% arterial.^6,11,25–27^ Two of the 12 named vessels in our study were arterial. A consistent decline was observed in vascular issues, with most of them occurring in the first quintile and none in the last quintile.

The complication rate from ALIF has been reported to be up to 40%.^28^ Our study showed 15% postoperative complications, both minor and major, as given in Table 4. The 30-day readmission/ED visit rate was 11%. The DVT rate was 2%, which is consistent with 0% to 5% reported in the literature.^6,25,27^ Low rates of DVT could be attributed to the use of handheld vascular retractors with frequent relaxation of pressure on vessels from retractors during the case (i.e., while obtaining fluoroscopic images). Other complications included delayed return of bowel function (2%), lower extremity swelling/pain (4.7%), superficial wound issues (5.5%), and urinary tract infection (0.8%). A single deep anterior infection with *Propionibacterium acnes* occurred in our study; there were no posterior infections. Perioperative mortality was consistent with that found by Garg et al^25^ of 0% (0% to 4% reported in the literature).

Higher BMI showed increased risk regarding case duration, EBL, and vascular complications. BMI >35 was associated with significant risk of vascular injury; the odds ratio was significant at 4.09 (1.4 to 11.7 CI, *P ≤* 0.008). Although not statistically significant, there was a trend toward higher postoperative complications in patients with BMI more than 35. Garg and Ballard et al. also emphasized the increased risk of conducting ALIFs in patients with higher BMI. Difficult exposure, poor visualization, and a deep wound are associated with higher complication rates in these patients.^25,29^ Careful patient selection and preoperative counseling are important to mitigate risks associated with the mini-ALIF procedure. This is in contrast to Rosen et al.^30^ who did not find increased complications associated with higher BMI in patients undergoing minimally invasive spine surgery; however, the authors only reviewed minimally invasive transforaminal lumbar interbody fusion (MIS-TLIF) procedures. We recommend a preoperative patient visit with the approach surgeon to discuss complications, review anatomy, and assess patient body habitus.

In addition, the hospital LOS displays a wide SD and can be best explained by the relative rarity of very few patients undergoing stand-alone mini-ALIFs during this study period. By 2022, our institution has advanced stand-alone mini-ALIF cases to be safely completed as an outpatient procedure.

Study limitations include the retrospective nature of this study and the lack of comprehensive data on the separate anterior and posterior components of the procedure. Other limitations include single-center, single-approach surgeon experience. It was also difficult to fully account for other predictors of perioperative and postoperative complications and analyze their variation between those who underwent the first versus 127th procedure, given that the study period spanned 9 years.

Armed with the information from this study, the authors have several recommendations for surgeons adopting the anterior approach. The incision should be carefully planned with fluoroscopy to ensure an ideal working corridor. If the surgeon has an opportunity to include a more experienced surgeon as an assistant for their initial cases, ideally, they will work together for the first 25 cases, which may help minimize complications during the learning period that has been established with our data. We have found that during certain cases, initial difficulty in mobilizing the major vessels becomes easier as the diskectomy proceeds, especially in cases where a spondylolisthesis is reduced. Because of this, in cases with difficult vessel mobilization, such as higher grade spondylolisthesis, we initiate the diskectomy and disk space preparation before fully mobilizing the vessels and then progressively mobilize the vessels and expand the annulotomy and associated disk space access. We have also learned that minor blood vessel injuries, which are small and usually relatively easily contained with application of focal pressure, may not need to be repaired. Try to apply local pressure with a sponge on a stick in a way the spine surgeon can complete the ALIF, and often when pressure is released, there is no ongoing bleeding and vascular repair is not necessary. Finally, the spine surgeon and approach surgeon should try to find opportunities to observe ALIF surgeries conducted by surgeons with substantial experience and consider spending time together in a cadaver laboratory.

## Conclusion

The mini-ALIF approach is a minimally invasive spine fusion technique that allows for disk height restoration and spondylolisthesis reduction with placement of a relatively large footprint interbody cage. Performance of the mini-ALIF approach is associated with a learning curve of 25 to 30 cases before complication rates begin to decline. Moreover, BMI > 35 is associated with increased surgical time and complication rate.
